# Filamin C promotes lymphatic invasion and lymphatic metastasis and increases cell motility by regulating Rho GTPase in esophageal squamous cell carcinoma

**DOI:** 10.18632/oncotarget.14087

**Published:** 2016-12-22

**Authors:** Kan Tanabe, Yoshinari Shinsato, Tatsuhiko Furukawa, Yoshiaki Kita, Kazuhito Hatanaka, Kentaro Minami, Kohichi Kawahara, Masatatsu Yamamoto, Kenji Baba, Shinichiro Mori, Yasuto Uchikado, Kosei Maemura, Akihide Tanimoto, Shoji Natsugoe

**Affiliations:** ^1^ Department of Digestive Surgery, Breast and Thyroid Surgery, Graduate School of Medical and Dental Sciences, Kagoshima University, Kagoshima, Japan; ^2^ Department of Molecular Oncology, Graduate School of Medical and Dental Sciences, Kagoshima University, Kagoshima, Japan; ^3^ Department of Molecular and Cellular Pathology, Graduate School of Medical and Dental Sciences, Kagoshima University, Kagoshima, Japan; ^4^ Center for the Research of Advanced Diagnosis and Therapy of Cancer, Graduate School of Medical and Dental Sciences, Kagoshima University, Kagoshima, Japan

**Keywords:** FLNC, ESCC, Rac1, Cdc42, migration

## Abstract

To establish treatments to improve the prognosis of cancer patients, it is necessary to find new targets to control metastasis. We found that expression of FilaminC (FLNC), a member of the actin binding and cross-linking filamin protein family is correlated with lymphatic invasion and lymphatic metastasis in esophageal squamous cell carcinoma (ESCC) by increasing cell motility through activation of Rho GTPase.

Immunohistochemistry analysis showed that FLNC expression in ESCC is associated with lymphatic invasion, metastasis, and prognosis. FLNC knockdown in esophageal cancer cell lines decreased cell migration in wound healing and transwell migration assays, and invasion in transwell migration assays. Furthermore, FLNC knockdown reduced the amount of activated Rac-1 (GTP-Rac1) and activated Cdc42 (GTP-Cdc42). Our results suggest that FLNC expression is a useful biomarker of ESCC metastatic tendency and that inhibiting FLNC function may be useful to control the metastasis of ESCC.

## INTRODUCTION

Invasion and metastasis are fundamental characters of cancer [1, 2]. The TNM classification of the International Union Against Cancer lists the major prognostic factors in cancer as tumor volume, depth of invasion, and presence of metastasis in lymph nodes or in distant organs [3]. Thus, cancer therapies focus on cancer cell invasion and mechanism of metastasis. However, the ability of cancer cells to migrate is an important element of cancer invasion and metastasis.

Cell migration is an actin-dependent process, involving the polymerization, de-polymerization, branching, and cross-linking of actin filaments [4]. Filamins are a family of actin binding and cross-linking proteins, originally extracted from chicken gizzard muscle [5]. Three highly conserved filamin isoforms have been identified in human: filamin A (FLNA; ENSG00000196924), filamin B (FLNB; ENSG00000136068), and filamin C (FLNC; ENSG00000128591) [6]. FLNA and FLNB are widely expressed in human tissues while FLNC is restrictively expressed in skeletal and cardiac muscle [7]. All three filamins cross-link actin filaments into three-dimensional structures, and link them to cellular membranes allowing them to serve as scaffolds for transmembrane receptors, channels, signaling molecules, and transcription factors [8, 9].

Several studies have described that FLNA and FLNB interact with a number of proteins to regulate signaling events involved in cell shape and migration in cancer [10–17]. In nasopharyngeal and gastric cancer, FLNA expression is correlated with poor prognoses [15, 16].

However, there are fewer reports associating FLNC with cancer, and the results are inconsistent [18–21]. To understand the oncological role of FLNC, we studied esophageal squamous cell carcinoma (ESCC). ESCC is the most common esophageal cancer in Asian countries [22], and is associated with poor prognosis due to the tendency of local invasion and metastasis [23, 24]. Therefore, local invasion and metastasis is one of the greatest obstacles of successful ESCC treatment.

Here, we performed immunohistochemistry (IHC) to evaluate FLNC protein expression in ESCC tissues and its relationship with clinicopathological factors. We then examined the function of FLNC in ESCC cells using short hairpin RNA (shRNA) interference. Given that Rho GTPase plays an important role in cell motility and the actin filament system, we examined the role of Rho GTPase in ESCC to elucidate how FLNC promotes cell motility. Our data indicate that FLNC expression is associated with lymphatic invasion and lymphatic metastasis and prognosis. Furthermore, we show that FLNC expression increases cell migration and invasion by regulating Rho GTPase.

## RESULTS

### Relationship between FLNC expression and clinicopathological findings

**Figure 1:**
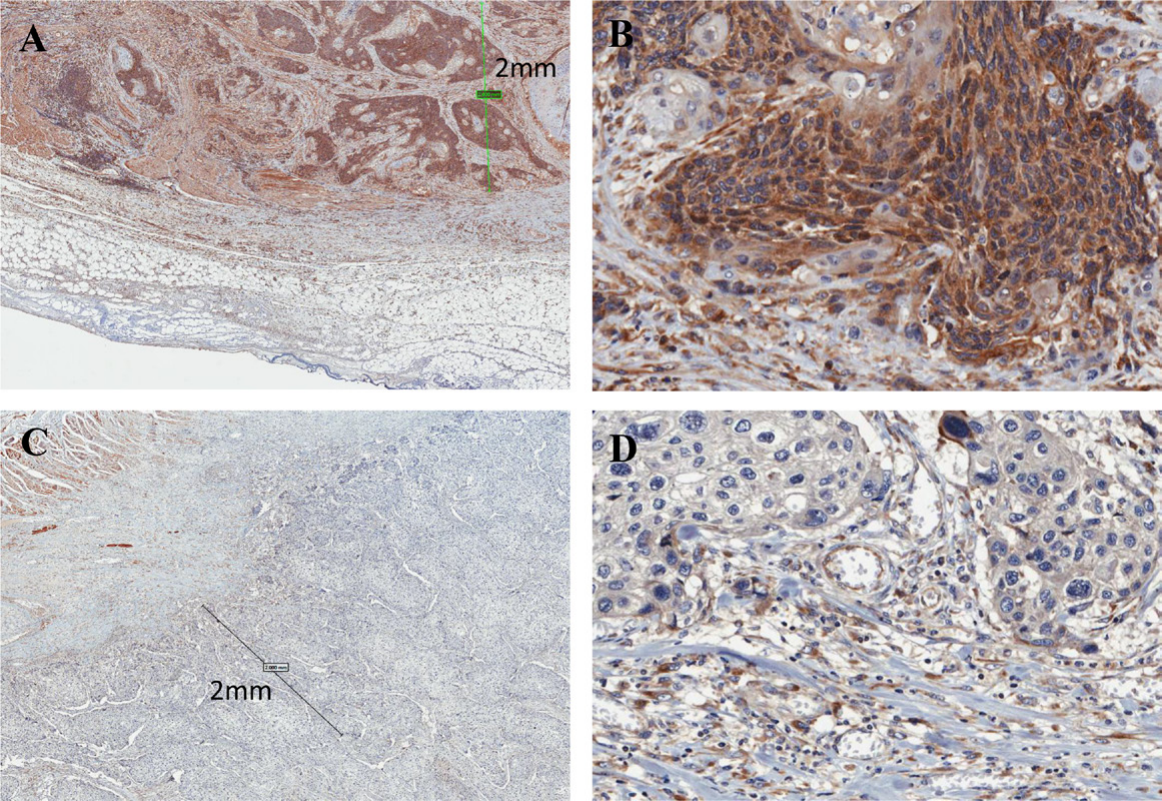
Immunohistochemistry of FLNC in ESCC tissues (**A**) High FLNC expression was detected in the cytoplasm ESCC marginal portion cells (within 2 mm of the invasive external edge of tumor). Scale bar indicates 2 mm (× 80); (**B**) magnified view (× 400). Images were captured by an Aperio CS2 scanner. (**C**) Low expression of FLNC (× 80); (**D**) magnified view (× 400).

To determine whether the expression of FLNC protein is associated with ESCC patient prognosis, we examined 75 cases of advanced ESCC without superficial carcinoma (Table 1). All patients had received radical surgery without neoadjuvant chemotherapy or radiotherapy. IHC analysis showed that FLNC is predominantly expressed in the cytoplasm of ESCC cells (Figure 1). The biological information of interactions between cancer and the extracellular matrix (ECM) is reflected by histological characteristics at the invasive front, rather than the body of the tumors [25]. Therefore, we evaluated FLNC expression in a marginal portion, within 2 mm of the invasive external edge of the tumors as described previously [26]. Patients were divided into two groups based on whether levels of FLNC expression were lower or higher than the average. Expression levels were measured as ratio of all patients and the percentages of the low and high FLNC expression groups were 30.7% (23/75 patients) and 69.3% (52/75 patients), respectively. FLNC expression was associated with lymphatic metastasis (N) (*P* = 0.032) and lymphatic invasion (ly) (*P* = 0.032). However, there were no significant associations between FLNC expression and age, gender, histological grade, depth of invasion (T), venous invasion (v), or pathological stage (pStage) (Table 1). However, FLNC expression and pStage trend to correlate (*p* = 0.051).

**Table 1:** Relationship between FLNC expression in ESCC and clinicopathologic findings

	Total *n* = 75	%	FLNC expression				*P*-value
			High *n* = 52 (69.3%)	%	low *n* = 23 (30.7%)	%	
Age (mean ± SD)	64.9 ± 8.99		65.3 ± 8.53		64.1 ± 10.11		NS
Gender							0.650
Male	61	81.3	43	82.7	18	78.3	
Female	14	18.7	9	17.3	5	21.7	
Histlogy							0.268
Well	32	42.7	20	38.5	12	52.2	
Moderate/Poor	43	57.3	32	61.5	11	47.8	
pT							0.702
pT2	11	14.7	8	15.4	3	13.0	
pT3/T4	64	85.3	44	84.6	20	87.0	
pN							0.032
pN0	23	30.7	12	23.1	11	47.8	
pN1/N2/N3	52	69.3	40	76.9	12	52.2	
Lymphatic invasion							0.032
Negative	23	30.7	12	23.1	11	47.8	
Positive	52	69.3	40	76.9	12	52.2	
Venous invasion							0.238
Negative	8	10.7	7	13.5	1	4.3	
Positive	67	89.3	45	86.5	22	95.7	
pStage							0.051
II	24	32.0	13	25.0	11	47.8	
III/IV	51	68.0	39	75.0	12	52.2	

NS: not significant.

Statistical analyses of two group differences were performed using the χ^2^-test. *P* < 0.05 was considered statistically significant.

### Relationship between FLNC expression and prognosis

**Figure 2:**
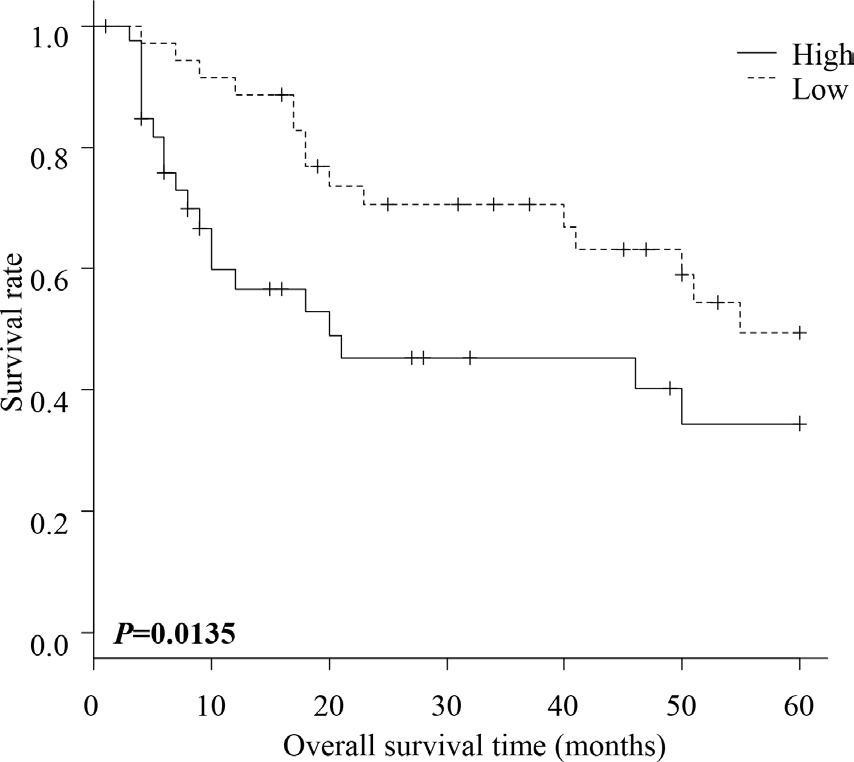
Overall survival of patients with ESCC Overall survival of ESCC patients within high and low FLNC expression groups. The patients in the high FLNC expression group had significantly poorer prognoses than those in the low FLNC expression group (*P* = 0.0135). Survival rates were calculated using the Kaplan Meier method and differences in survival were estimated by Wilcoxon test.

Disease free survival was not significantly different between patients with high and with low FLNC expression (Supplementary Figure S1). However, overall survival of patients in the high FLNC expression group was significantly shorter than the survival of patients in the low FLNC expression group (*P* = 0.0135) (Figure 2). These data indicate a correlation between high FLNC expression and poor prognosis in patients with ESCC.

### Univariate and multivariate analyses of survival

Univariate analysis showed that the significant prognostic variables for postoperative survival were ly (*P* = 0.009), pStage (*P* = 0.002), and FLNC expression (*P* = 0.047) (Table 2). Multivariate analysis using the above five parameters indicated that only ly (*P* = 0.018) was an independent prognostic factor. FLNC expression was not an independent prognostic factor.

**Table 2:** Univariate and multivariate analysis in patients with ESCC for overall survival (Cox regression analysis)

	Univariate analysis	Multivariate analysis		
	*P*-value	HR	95% CI	*P*-value
Gender	0.366	-	-	-
Histrogical grade (well/ mode, poor)	0.485	0.803	0.39–1.65	0.551
pT	0.202	2.508	0.86–7.34	0.093
pN	0.063	-	-	-
Lymphatic invasion	0.009	2.942	1.20–7.19	0.018
Venous invasion	0.218	0.618	0.25–1.52	0.293
pStage	0.002	-	-	-
FLNC expression (High/ Low)	0.047	0.497	0.22–1.11	0.088

HR: hazard ratio, CI: confidence interval.

Univariate analyses and multivariate analysis were performed using Cox regression analysis. *P* < 0.05 was considered statistically significant.

### Generation of FLNC knock-down cells

**Figure 3:**
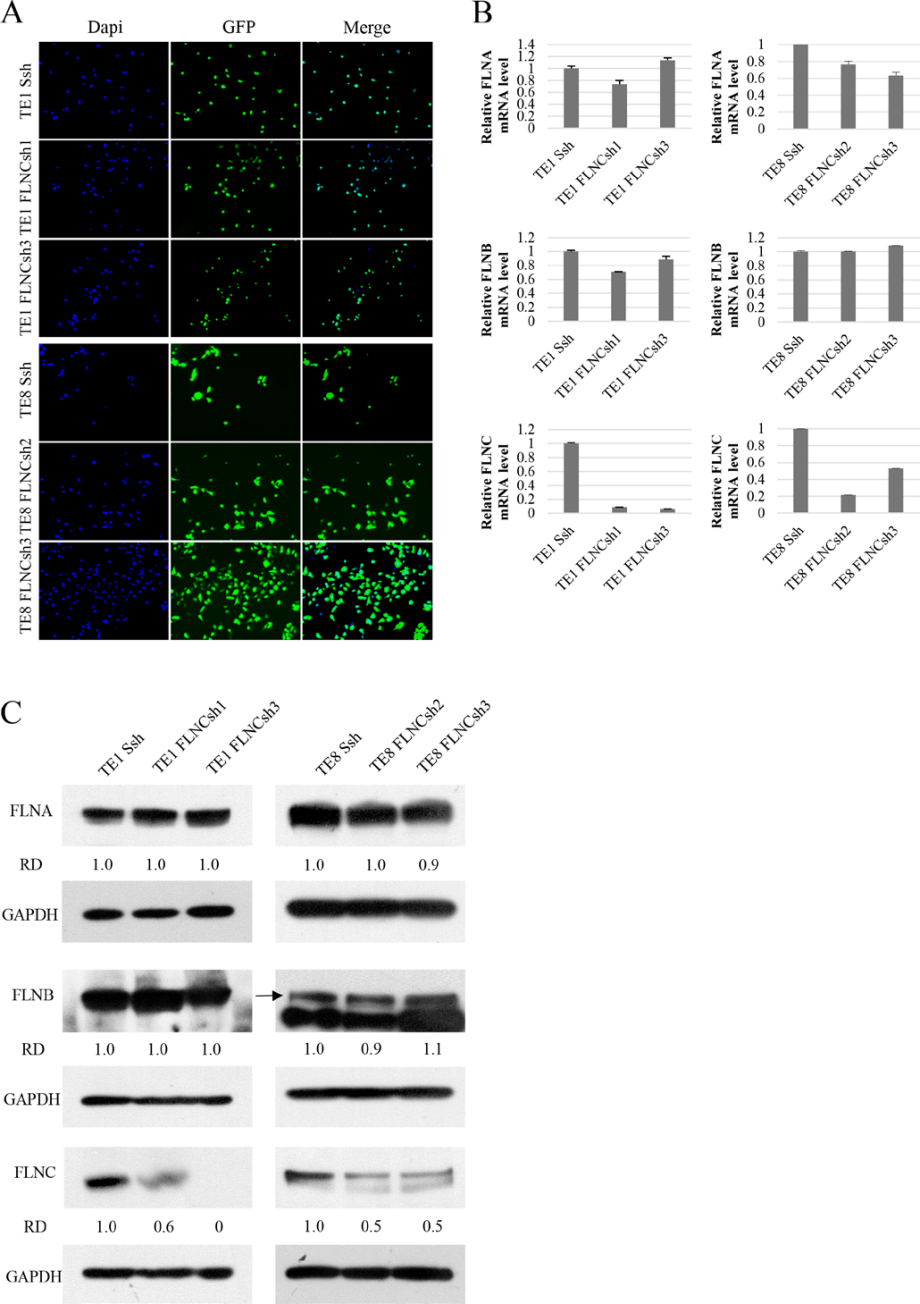
shRNA of FLNC specifically inhibits FLNC expression in shRNA infected ESCC cell lines (**A**) Infection efficiency of shRNA lentiviral vectors in the ESCC cell lines (TE-1 and TE-8). Infection efficiency of shRNA lentiviral vectors was confirmed by visualization of green fluorescent protein (GFP) with a confocal microscope. Nuclei are stained with DAPI to visualize the cells. More than 90% of cells were confirmed to be infected with shRNA lentivirus. (**B**, **C**) mRNA and protein expression levels of FLNA, FLNB, and FLNC were examined by real-time PCR and immunoblotting. GAPDH was used as a loading control. The FLNA and FLNB expression were not reduced in FLNC shRNA infected cells, and were nearly identical to those of SshRNA infected cells. FLNC expression was clearly inhibited in FLNC shRNA infected cells as compared with its expression in SshRNA infected cells. Densities of the immunoblot bands were quantified using Image J software and normalized to GAPDH to obtain the relative densities (RD).

To examine the functions of FLNC, we first established FLNC knockdown ESCC cell lines from TE-1 and TE-8 cells using lentiviral delivery of shRNA. To determine the specific effect of FLNC knockdown, we selected FLNC shRNAs that do not affect the expression of FLNA and FLNB in each cell line. TE-1 cells were infected with lentivirus containing either Scrambled shRNA (SshRNA) or FLNC shRNA (FLNCsh1 and FLNCsh3), TE-8 cells were infected with lentivirus containing either SshRNA or FLNC shRNA2 and FlNCshRNA3. After selection of infected cells, GFP positive cells accounted for more than 90% of cells per microscope field (×100), for each shRNA (Figure 3A). We evaluated the expression of FLNA, FLNB, and FLNC using real-time PCR and immunoblotting (Figure 3B and 3C). FLNA and FLNB expression levels in FLNC shRNA infected cells were almost equivalent to those in SshRNA infected cells, indicating that FLNC shRNA specifically inhibited FLNC expression.

### Increased ESCC cell migration and invasion upon FLNC expression

**Figure 4:**
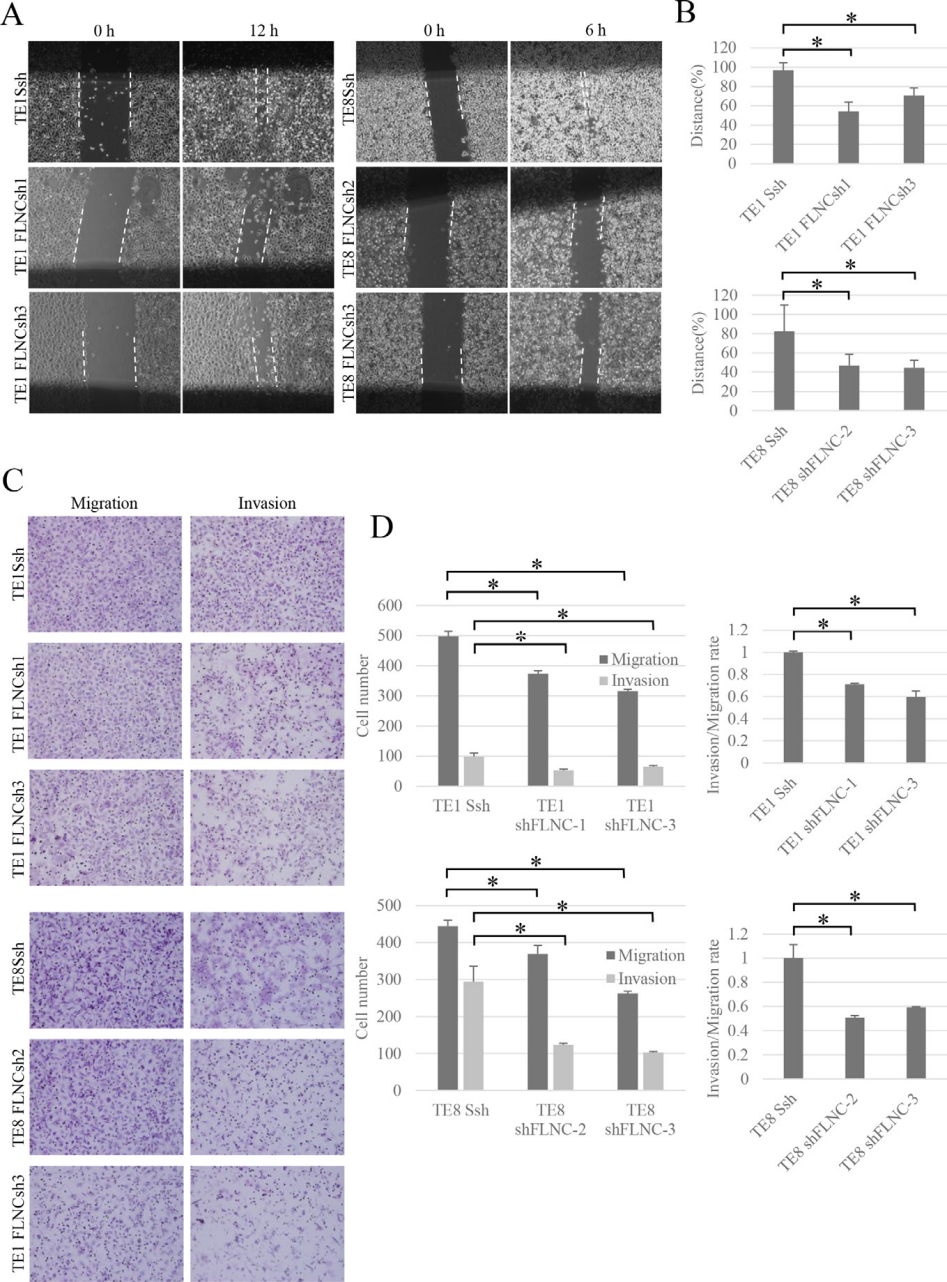
FLNC knockdown reduces cell migration and invasion activity (**A**) Effect of FLNC knockdown in wound healing assay (× 40). The pictures show cells at 12 hours in TE-1 linage and 6 hours in TE-8 linage after scraping. The dashed lines indicate the border of the cell free area. (**B**) The bar graphs indicate the widths of the cell free area of each cell type. FLNC knockdown inhibited cell migration significantly in both TE-1 and TE-8 cells. Columns represent three independent replicates and bars indicate SD. *P < 0.01, significantly different from SshRNA infected cells. (**C**) The transwell migration and invasion assay (× 200) was used to examine the effect of FLNC knockdown on cell migration and invasion activity. (**D**) Quantification of migration and invasion abilities of FLNC knockdown cells. The numbers of migratory and invasive cells in the FLNC knockdown cells were significantly less than in control cells. The invasion/migration rate of FLNC knockdown cells was also significantly less than the control cells. Columns represent total cell number in five independent microscopic fields and bars indicate SD. *P < 0.01, significantly different from the number of SshRNA infected cells.

We compared cells with FLNC shRNA mediated FLNC knockdown with SshRNA infected cells as control cells. The MTT assay showed that the proliferative abilities of TE-1 and TE-8 FLNC knock-down cells were almost same as those of the control cells (Supplementary Figure S2). Wound healing assays show that the width of the wounds in FLNC knock-down cells was much wider than in the controls after 12 or 6 hours (Figure 4A and 4B). Additionally, FLNC knockdown decreased the number of migratory and invasive cells that passed through the chamber in transwell migration and invasion assays (Figure 4C) Moreover, FLNC knockdown decreased invasion/migration rate. These data indicate that FLNC knockdown decreased the migratory and invasive abilities of cancer cells (Figure 4D).

### Decrease of activated Rho GTPase following FLNC knockdown

**Figure 5:**
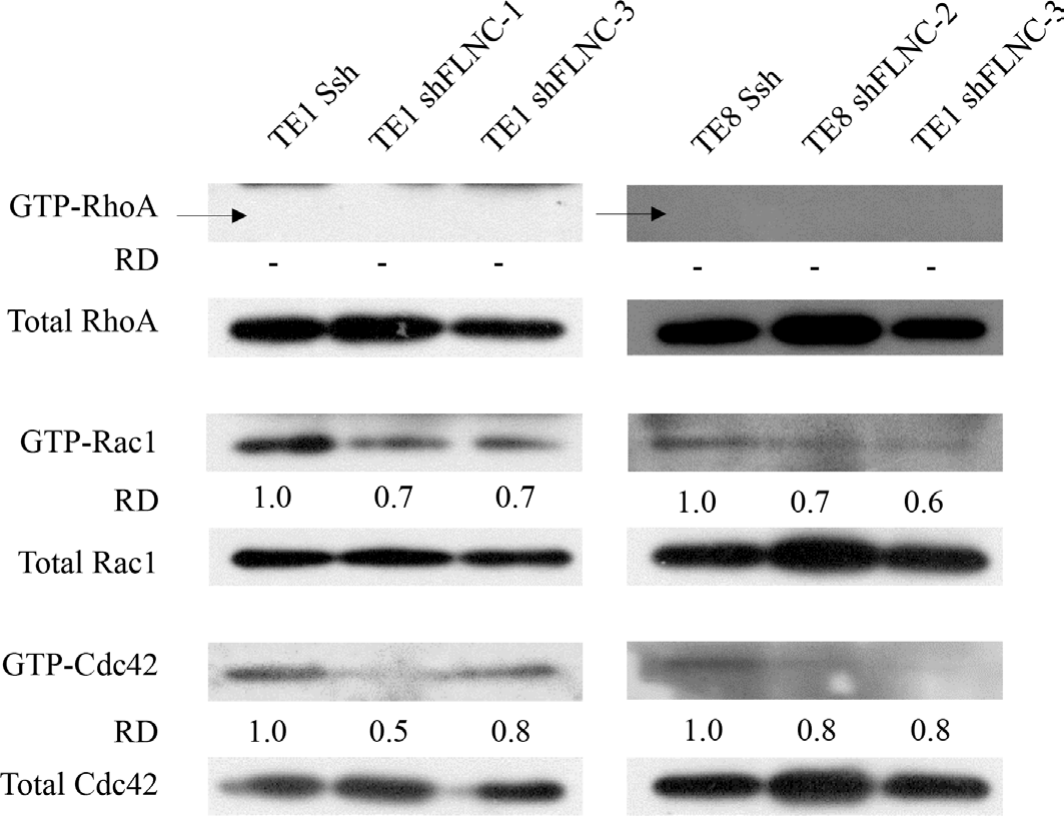
FLNC knock-down downregulates Rac1 and Cdc42 activation Rho GTPase activities were evaluated in total lysates by activated GTPase pull-down. FLNC knockdown was associated with suppression of GTP-Rac1 and GTP-Cdc42, but not GTP-RhoA in both TE-1 and TE-8 cells. Densities of the individual bands were quantified using Image J software. The densities of GTP-forms bands were normalized to the densities of bands of each total Rho family protein. Relative densities (RD)were obtained by comparing to the density of each GTP-form band of Ssh infected cells.

Rho family members play important roles in regulating cytoskeletal dynamics. Therefore, we hypothesized that FLNC decreses Rho-mediated cell motility. To investigate how FLNC knockdown inhibits ESCC cell motility, we examined Rho GTPase activation (Figure 5). The levels of activated Rac1 (ENSG00000136238) (GTP-Rac1) and Cdc42 (ENSG00000070831) (GTP-Cdc42) were clearly reduced in FLNC knockdown cells compared with SshRNA infected cells. We could not detect GTP-RhoA (ENSG00000067560) in either TE-1 or TE-8 cells.

## DISCUSSION

Numerous studies have reported that expression of FLNA and FLNB promote cancer invasion and metastasis [12–17]. Meanwhile, the role of FLNC in cancer remains controversial [18–21]. FLNC expression in glioma has been reported to increase with advancing tumor grade, and serum anti-FLNC autoantibody can be a potential biomarker for early glioma diagnosis [19]. However, the expression of FLNC in prostate and gastric cancer was lower than in normal tissues and overexpression of FLNC reduced the invasive abilities of these cell lines [21]. Moreover, in prostate, leukemia, and breast cancer, high expression of FLNC mRNA has been associated with better prognosis by *‘in silico’* analysis [21]. The conclusions of these reports are inconsistent and the role of FLNC in cancer remains unclear.

Biological information about cancer and interactions between cancer and the ECM is reflected by histological characteristics at the invasive front of the lesion rather than in the body of the tumor [25]. Therefore, we assessed FLNC expression in marginal portion of the tumors, within 2 mm of the invasive external edge of the tumor [26]. We observed that FLNC expression was significantly associated with lymphatic metastasis, lymphatic invasion, and clinical stage. Moreover, ESCC patients with high FLNC expression had poorer prognosis, consistent with the fact that lymphatic metastasis is a serious prognostic factor for ESCC [23]. We propose that FLNC expression affects the prognosis of ESCC patients by promoting lymphatic invasion and lymphatic metastasis. Our clinical data is similar to results observed for FLNC expression in glioma tissues, but differs from those observed in prostate cancer, breast cancer, and leukemia [19–21].

We then examined the role of FLNC in ESCC cell lines with have high levels of FLNC expression. We knocked-down FLNC expression in ESCC cells using shRNA. Wound healing assays and transwell migration and invasion assays showed that FLNC promoted cell migration and invasion in ESCC. Interestingly, the opposite has been reported for gastric and prostate cancer cells, where FLNC expression inhibited cell migration and invasion [21]. We consider that the role of FLNC may differ depending tissue and/or tumor type.

Our results indicate that the function of FLNC resembles that of FLNA. FLNA promotes cancer cell migration and invasion [11, 12, 27], and regulates actin remodeling by activating Rho GTPase [28, 29]. Rho GTPase plays an important role in regulating the organization of the actin filament system [30–33]. GTPase family members have two conformations, an active GTP-bound conformation, and an inactive GDP-bound conformation. Rho GTPase activation assays showed that FLNC knockdown reduced GTP-Rac1 and GTP-Cdc42 levels. Rac1 is involved in the formation of membrane ruffles and the protrusions at the leading edge of migrating cells, lamelipodia [34]. Cdc42 is involved in the formation of filopodia, which function to probe the surrounding environment and control the direction of cell migration [32, 34, 35]. RhoA also leads to cell motility by maintaining focal adhesions and in the assembly of stress fibers [32, 34]. However, GTP-RhoA expression was too low to detect in both TE-1 and TE-8 cells in our assays. Therefore, it is possible that Rac1 and Cdc42 activation are the driving forces of FLNC dependent ESCC cell motility. Several studies indicate that Rho GTPase is associated with lymphatic metastasis [36–38]. These reports support the correlation between our clinical and *in vitro* data. Therefore, FLNC potentially promotes lymphatic invasion and lymphatic metastasis by regulating Rac1 and Cdc42 activity in ESCC.

Matrix metalloproteinases (MMPs) are a family of structurally related zinc-dependent endopeptidases that are often increased in the tumor microenvironment and able to degrade various components of the ECM and lead to cancer cell invasion and metastasis [39–42]. MMPs also enhance the progression of the epithelial-to-mesenchymal transition (EMT) [43, 44]. Therefore, MMPs are often associated with a poor prognosis of cancer patients [39, 45, 46]. MMPs are associated with clinicopathologic factors and prognosis in ESCC [47–49]. In addition, MMPs are also reported to be associated with Rho GTPase in cell invasion [50–52]. MMPs are expected to be associated with FLNC-Rac1 and cdc42 axis and promote the invasion of ESCC in this study.

Our studies show that high FLNC expression in ESCC patients is correlated with lymphatic invasion, lymphatic metastasis, and an unfavorable prognosis. Additionally, we demonstrate that FLNC knockdown inhibited ESCC cell migration and invasion, possibly by regulating Rho GTPase. Our results suggest that FLNC might be a useful biomarker, and a promising therapeutic target, of lymphatic metastasis in ESCC patients. Future studies are required to define the mechanisms by which FLNC activates cell migration and invasion in ESCC.

## MATERIALS AND METHODS

### Drugs, reagents, and antibodies

The following reagents were purchased from the indicated manufacturers:

RPMI 1640 (Nikken Biomedical Laboratory, Osaka, Japan); fetal calf serum (FCS) (PAA Laboratories, Pasching, Austria); MTT (3-(4,5-dimethylthiazol-2-yl)-2,5-diphenyl tetrazolium bromide) (Sigma-Aldrich, St. Louis, MO, USA); DAPI (4′,6-Diamidino-2-phenylindole, dihydrochloride, solution) (Dojindo Laboratories, Kumamoto, Japan); monoclonal antibodies against FLNA, GAPDH (EMD Millipore, Billerica, MA, USA; Cell Signaling Technology, Danvers, MA, USA, respectively), polyclonal antibodies against FLNB, FLNC (EMD Millipore, Billerica, MA, USA; Atlas Antibodies, Stockholm, Sweden, respectively).

### Patients and tumor samples

The study included 75 consecutive patients with advanced ESCC (T2-4) who underwent surgical treatment in the Department of Digestive Surgery and Breast and Thyroid Surgery of Kagoshima University Hospital between January 2005 and January 2009. The clinical samples were obtained from tumors that were surgically removed and pathologically confirmed ESCC. The patients included 61 men and 14 women who ranged in age from 38 to 79 years old. None of the patients received preoperative radiotherapy or chemotherapy. The pathological features of ESCC were defined according to the TNM classification [3]. The study was approved by the Institutional Review Board of Kagoshima University, and performed in accordance with the Helsinki Declaration. Informed consent was obtained for each patient.

### Immunohistochemical analysis of patient tumors

Surgical samples were fixed in 10% formaldehyde and embedded in paraffin before being cut into 3-μm slices. Deparaffinization, hydrophilization, and target retrieval were performed in the PT Link system (Dako, Denmark). Endogenous peroxidase activity was blocked with 3% hydrogen peroxide in methanol. After washing three times with PBS, the sections were preincubated in 1% bovine serum albumin for 30 minutes to block nonspecific reactions. The sections were incubated with FLNC rabbit polyclonal antibody (1:100 dilution) as the primary antibody overnight. Staining was performed using the avidin-biotin complex and immunoperoxidase method (Vectastatin ABC Kit, Vector Laboratories, Inc., Burlingame, CA, USA). The sections were visualized using diaminobenzidine tetraydrochloride. Images were captured using an Aperio CS2 scanner (Leica Biosystems, Nussloch, Germany). We evaluated FLNC expression in the marginal portions of the tumors, defined as within 2 mm in diameter of the invasive external edge of the tumor [26]. The number of cells in five microscopic fields (magnification × 200) was counted independently by two researchers (K.T and Y.K). Positive cells were counted and ratios were obtained by dividing the number of immunopositive cells by the total number of cancer cells per field, and are expressed as a percentage.

### Cells and cell culture

The human ESCC cell lines TE-1 and TE-8 were obtained from RIKEN BioResource Center (Tsukuba, Japan). Cells were cultured in RPMI 1640 supplemented with antibiotics (100 U / mL penicillin) and 10% FCS. All cancer cell lines were cultured at 37°C in a 5% CO_2_ humidified atmosphere.

### FLNC mRNA interference

pENTR4-H1, CS-RfA-CG was obtained from RIKEN BioResource Center, Dr. Hiroyuki Miyoshi (Tsukuba, Japan). Oligonucleotides for FLNC protein suppression by shRNA (shRNA1, shRNA2, and shRNA3) interference were designed using siDirect (http://sidirect2.rnai.jp/) and obtained from FASMAC (Kanagawa, Japan). Sequences are provided in Supplementary Table S1. These oligonucleotides and Scrambled shRNA (SshRNA) [53] were annealed and ligated into the pENTR4-H1 entry vector using BglII and XbaI enzyme sites, respectively. The resulting pENTR4-H1-FLNC-shRNA1, pENTR4-H1-FLNC-shRNA2, pENTR4-H1-FLNC-shRNA3, or pENTR4-H1-SshRNA vectors and the CS-RfA-CG destination vector were used in a recombination reaction using LR Clonase II (Invitrogen, Carlsbad, CA, USA) according to the manufacturer's instructions. 293FT cells (Invitrogen, Carlsbad, CA, USA) were plated in 6-well plates and transfected with the lentiviral vector plasmid (CS-FLNC-shRNA1-CG, CS-FLNC-shRNA2-CG, CS-FLNC-shRNA3 or CS-SshRNA-CG) and lentivirus packaging plasmids (pMDLg/pRRE, pRSV-Rev, pMD2.G (Addgene, Cambridge, MA, USA)) using Lipofectamine 2000 (Invitrogen, Carlsbad, CA, USA) at 37°C for 48 hours. The supernatants, containing lentivirus, were concentrated to 10 times using Lenti-X™ Maxi Purification Kit (Clontech, Shiga, Japan). The ESCC cell lines plated in 6-well plates were infected by lentivirus at 37°C for 48 hours. After infection, cells were inoculated in a 10-cm^2^ dish (500 cells / dish) for cloning. After seven days, colonies clearly derived from a single cell were selected based on GFP fluorescence.

### Cell proliferation assay

Equal numbers of cells (1 × 10^3^) were inoculated into each well and incubated for 1, 3, 5, and 7 days. Cell viability was measured using the MTT colorimetric assay [54].

### Wound healing assay

A total of 4 × 10^5^ cells were seeded in 12 well plates. After overnight incubation, cells were scraped with a 200-μL pipette tip to make a straight-line cell-free scratch. Each well was washed with PBS to remove the remaining unattached cells. The cells were observed and pictures were taken 12 hours (TE-1 linage) and 6 hours (in TE-8 linage) after scraping. Cell motility was quantified by measuring the distance between the migrating cell boundaries.

### Transwell migration and invasion assay

Migration and invasion assays were performed using transwell chambers. For invasion assays, we used BioCoat^TM^ Matrigel Invasion Chamber 24-well 8 μm (Corning, NY, USA). Cells (1 × 10^5^) were seeded with serum free medium onto the top chamber, and the bottom chamber was filled with medium including 10% FCS and 1 ng/mL EGF (PeproTech, Rocky Hill, NJ, USA). For the migration assays, we used BioCoat^TM^ Control Inserts 24 well 8 μm. A chamber membrane was coated with 10 μg/mL fibronectin (Sigma-Aldrich, St. Louis, MO, USA). After 48 hours of seeding the cells, the bottom membranes were fixed using 4% paraformaldehyde, and stained with hematoxylin. Cell numbers were quantified by counting 5 random fields under a microscope at a magnification of 200 ×.

### RNA isolation and cDNA synthesis

Total RNA from the cultured cells was isolated using TRIzol (Invitrogen, Carlsbad, CA, USA), and reverse-transcribed using the ReverTra Ace kit (Toyobo, Osaka, Japan), according to the manufacturer's instructions [55].

### Quantitative real-time PCR

mRNA expression levels of FLNA, FLNB, and FLNC were determined by real-time RT-PCR (Step One Plus^™^; Applied Biosystems, Foster City, CA, USA) using Go Taq qPCR Master Mix (Promega, Wisconsin, USA) according to the manufacturer's instructions. Human GAPDH was used for normalization. The expression of the target gene was quantified using the comparative cycle threshold method. The respective forward and reverse primer sequences are provided in Supplementary Table S2.

### Protein extraction and immunoblotting

Total cell lysate was isolated with RIPA buffer (25 mM Tris-HCl (pH 7.5), 150 mM NaCl, 1% Nonidet P-40 (NP-40), 0.1% SDS, 0.5% sodium deoxycholate, 1 mM p-amidinophenyl methanesulfonyl fluoride hydrochloride (APMSF), and 1 μg/mL aprotinin). Protein concentrations were measured using the BioRad protein assay kit (Hercules, CA, USA). Cell lysates (20 μg protein for FLNA, and 50 μg protein for FLNB and FLNC) were separated by 5% – 20% SDS-polyacrylamide gel (ATTO, Tokyo, Japan) electrophoresis and transferred onto membranes as described previously [56]. The blotted membranes were incubated with anti-FLNA (1:500000 dilution), anti-FLNB (1:1000 dilution), anti-FLNC (1:500 dilution), and GAPDH (1:100000 dilution) antibody overnight at 4°C, and each protein was detected as described previously [56]. Protein expression was quantified using Image J software (National Institutes of Mental Health, Bethesda, MD, USA) and normalized to GAPDH to obtain the relative densities (RD).

### Small GTPase pull-down assay

Small GTPase pull-down assay was performed using the RhoA/Rac1/Cdc42 Activation Assay Combo Kit (Cell BioLabs, San Diego, CA, USA) following the manufacturer's instructions. Cells were incubated with medium and 1 ng/mL EGF for 2–4 days until they obtained approximately 80–90% confluence. Cell lysates were incubated with Rhotekin RBD (for RhoA) or PAK1 PBD (for Rac1/Cdc42) agarose beads for 1 hour at 4°C. GTP-RhoA, GTP-Rac1, or GTP-Cdc42 was detected by immunoblotting.

### Statistical analysis

All of the statistical calculations were carried out with EZR (http://www.jichi.ac.jp/saitama-sct/) [57]. Statistical analyses of group differences were performed using the χ^2^-test and the Student's *t*-test. Patients were divided into FLNC expression high and low groups based on median FLNC expression levels. Kaplan-Meier survival curves were generated comparing these two groups via the Wilcoxson test. Univariate analysis and multivariate analysis were evaluated by Cox regression analysis. Differences were considered significant at *P* < 0.05.

## SUPPLEMENTARY MATERIALS FIGURES AND TABLES


