# Supplementary material for: Filamin C promotes lymphatic invasion and lymphatic metastasis and increases cell motility by regulating Rho GTPase in esophageal squamous cell carcinoma
Source: Oncotarget. 2016 Dec 22;8(4):6353–63. doi: 10.18632/oncotarget.14087 (PMC5351637; doi:10.18632/oncotarget.14087)
Supplement: Supplementary file 1 [file oncotarget-08-6353-s001.pdf]

Filamin C promotes lymphatic invasion and lymphatic metastasis and increases cell motility by regulating Rho GTPase in esophageal squamous cell carcinoma

Supplementary Materials

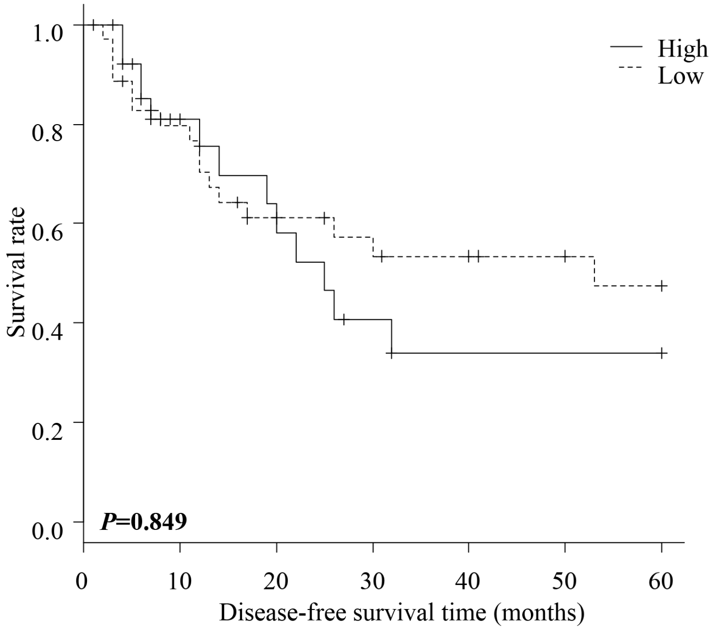

Supplementary Figure S1: Disease free survival of patients with ESCC Disease free survival of ESCC patients within high and low FLNC expression groups. There was no statistically significant difference observed. Survival rates were calculated using the Kaplan Meier method and differences in survival were estimated by Wilcoxon test.

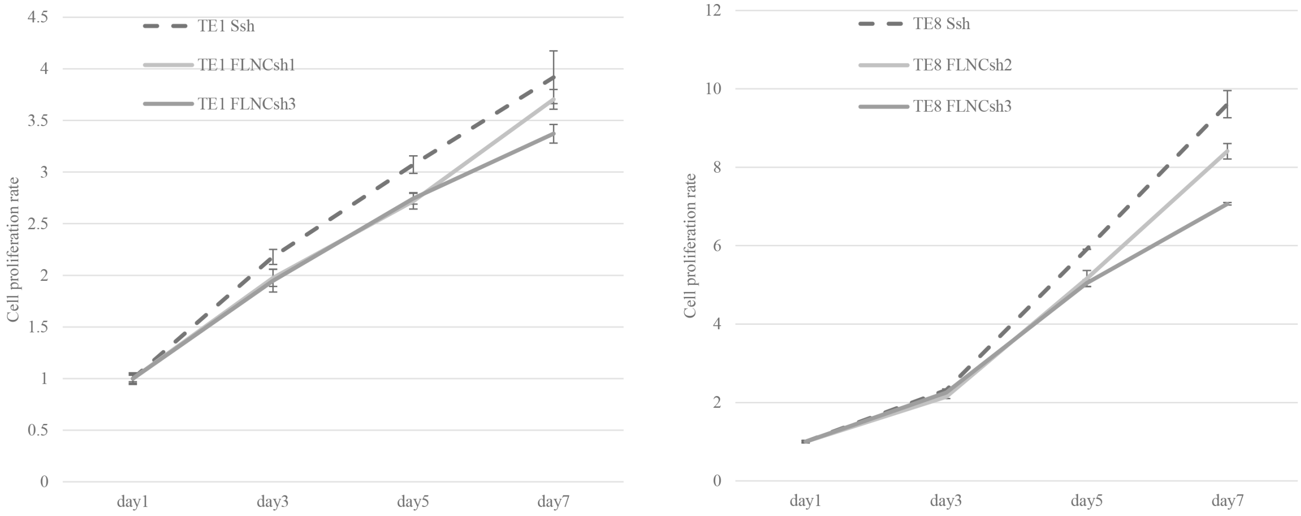

Supplementary Figure S2: Cell proliferation assay of shRNA infected ESCC cell lines Equal numbers of cells (1×10^3) were inoculated into each well and incubated for 1, 3, 5, and 7 days. Cell viability was measured using the MTT assay.

Supplementary Table S1: FLNC shRNAs sequences

Construct name	sequences
FLNC shRNA1	(sense) 5′-GATCCCCGTACCTTTGACATCTACTACAACGTGTGCTGTCCGTTGTAGTAGATGTCAAAGGTACTTTTTGGAAAT-3′<br>(antisense) 5′-CTAGATTTCCAAAAAGTACCTTTGACATCTACTACAACGGACAGCACACGTTGTAGTAGATGTCAAAGGTACGGG-3′
FLNC shRNA2	(sense) 5′-GATCCCCGACTTCAAGGTGTTTACCAAGACGTGTGCTGTCCGTCTTGGTAAACACCTTGAAGTCTTTTTGGAAAT-3′<br>(antisense) 5′-CTAGATTTCCAAAAAGACTTCAAGGTGTTTACCAAGACGGACAGCACACGTCTTGGTAAACACCTTGAAGTCGGG-3′
FLNC shRNA3	(sense) 5′-GATCCCCGATTACGTCCTCGTTGTCAACGTGTGCTGTCCGTTGACAATGAGGATGTAGTCTTTTTGGAAAT-3′<br>(antisense) 5′-CTAGATTTCCAAAAAGACTACATCCTCATTGTCAACGGACAGCACACGTTGACAACGAGGACGTAATCGGG-3′

Supplementary Table S2: The human FLNs Primers and GAPDH primers

Construct name	primer pair
FLNA	(forward primer) 5′-GCAACCTGACGGAGACCTAC-3′<br>(reverse primer) 5′-TGAACTTGTTGGGCTTGTTG-3′
FLNB	(forward primer) 5′-AACAGCACGGCCAAGTTCAT-3′<br>(reverse primer) 5′-CTTGCCCACGAGACCACCTT-3′
FLNC	(forward primer) 5′-AGTGGCGTCAAGGTCTCAGG-3′<br>(reverse primer) 5′-GCGGTGTACTGCACTCGGTA-3′
GAPDH	(forward primer) 5′-TGCACCACCAACTGCTTAG-3′<br>(reverse primer) 5′-GAGGCAGGGATGATGTTC-3′
